# Supplementary material for: Goldilocks Dilemma: LPS Works Both as the Initial Target and a Barrier for the Antimicrobial Action of Cationic AMPs on E. coli
Source: Biomolecules. 2023 Jul 20;13(7):1155. doi: 10.3390/biom13071155 (PMC10377513; doi:10.3390/biom13071155)
Supplement: Supplementary file 1 [file biomolecules-13-01155-s001.zip › biomolecules-2492194-supplementary.pdf]

Supplementary material for:

Goldilocks Dilemma: LPS Works Both as the Initial Target and a Barrier for the Antimicrobial Action of Cationic AMPs on *E. coli*

Martin Jakubec [1,*], Fredrik G. Rylandsholm [1], Philip Rainsford [1], Mitchell Silk [1], Maxim Bril'kov [2], Tone Kristoffersen [1], Eric Juskewitz [3], Johanna U. Ericson [3] and John Sigurd M. Svendsen [1]

1 Department of Chemistry, Faculty of Science and Technology, UiT the Arctic University of Norway, 9019 Tromsø, Norway; fredrik.g.rylandsholm@uit.no (F.G.R.); philip.rainsford@uit.no (P.R.); tone.kristoffersen@uit.no (T.K.); john-sigurd.svendsen@uit.no (J.S.M.S.)

2 Department of Pharmacy, Faculty of Health Sciences, UiT the Arctic University of Norway, 9019 Tromsø, Norway; maxim.brilkov@uit.no

3 Department of Medical Biology, Faculty of Health Sciences, UiT the Arctic University of Norway, 9019 Tromsø, Norway; johanna.e.sollid@uit.no (J.U.E.)

* Correspondence: martin.jakubec@uit.no

Peptide chemical analysis

The crude cyclic peptides were purified by preparative reverse phase-HPLC using gradients from 15 to 40 % buffer B (buffer A: water/0.1% TFA, buffer B: acetonitrile/0.1% TFA) over 50 minutes (11 ml/min flow rate). c(LWwNKr) was isolated as a white fluffy solid. **HRMS** (ESI): Calculated for: $C_{44}H_{62}N_{13}O_7^+$ $[M+H]^+$ 884.4890; found; 884.4885. c(WWWRRR) was isolated as a white solid. **HRMS** (ESI): Calculated for: $C_{51}H_{68}N_{18}O_6^{2+}$ $[M+H]^{2+}$ 514.2779; found; 514.2775. c(WRWRWR) was isolated as a white solid. **HRMS** (ESI): Calculated for: $C_{51}H_{68}N_{18}O_6^{2+}$ $[M+H]^{2+}$ 514.2779; found; 514.2773. c(WWWKKK) was isolated as a white solid. **HRMS** (ESI): Calculated for: $C_{51}H_{68}N_{12}O_6^{2+}$ $[M+H]^{2+}$ 472.2687; found; 472.2682. c(WKWKWK) was isolated as a white solid. **HRMS** (ESI): Calculated for: $C_{51}H_{68}N_{12}O_6^{2+}$ $[M+H]^{2+}$ 472.2687; found; 472.2681.

Table S1: K_P and k_{off} from Figure 2.

AMP	K_P			k_{off} (s^{-1})		
	DMPC	*DMPC:PG*	*DMPC:LPS*	*DMPC*	*DMPC:PG*	*DMPC:LPS*
**LWwNKr**	278 ± 8	401 ± 19	1145 ± 99	1.76 ± 0.12	1.75 ± 0.16	1.01 ± 0.18
**WKWKWK**	531 ± 10	630 ± 33	1191 ± 166	0.90 ± 0.24	1.32 ± 0.05	0.95 ± 0.12
**WRWRWR**	1299 ± 94	3160 ± 15	4520 ± 215	0.87 ± 0.19	0.48 ± 0.05	0.70 ± 0.16
**WWWKKK**	2534 ± 80	5156 ± 34	3943 ± 218	0.48 ± 0.07	0.32 ± 0.05	0.60 ± 0.15
**WWWRRR**	6649 ± 80	12705 ± 16	17040 ± 1150	0.22 ± 0.02	0.19 ± 0.01	0.25 ± 0.03

Table S2: γB/γF-1 ratios.

AMP	γB/γF-1						
	DMPC	*DMPC:LPS*	*E. coli ATCC 25922 lysate*	*E. coli ATCC 25922*	*E. coli CCUG 70662-*	*E. coli CCUG 70662+*	*E. coli NR 698*
**LWwNKr**	7.830	2.620	0.035	0.106	5.202	4.990	0.186
**WKWKWK**	7.127	3.915	0.044	0.083	0.170	0.295	0.180
**WRWRWR**	2.900	3.475	0.275	0.143	0.549	1.062	3.059
**WWWKKK**	0.297	0.402	0.225	0.289	0.317	0.197	0.370
**WWWRRR**	0.247	0.199	0.216	0.239	0.473	0.238	0.819

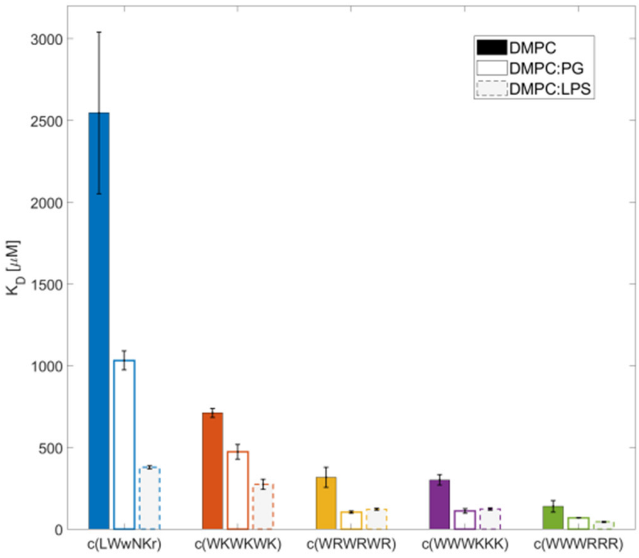

Figure S1: K_D extracted from steady state SPR analysis

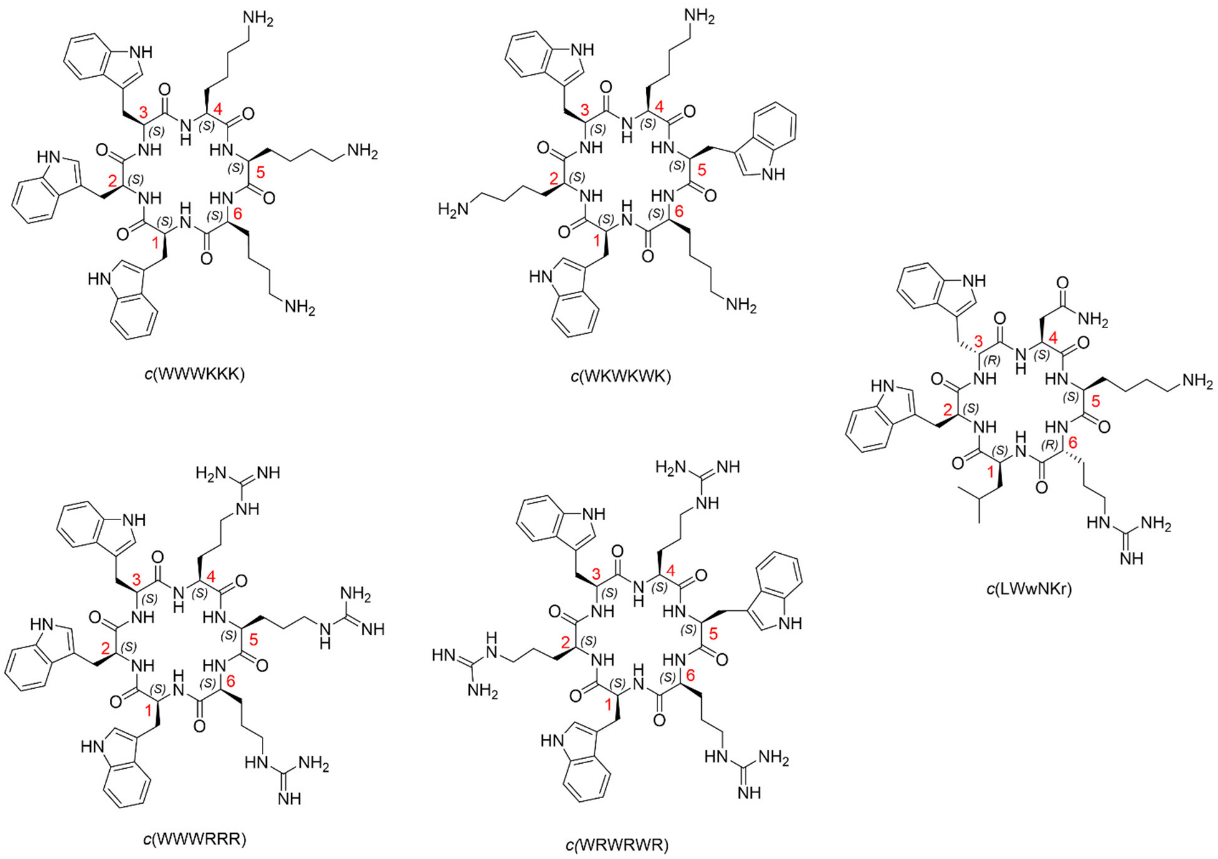

<u>Figure S2</u>: The five peptides with numbered amino acids for structure elucidation.

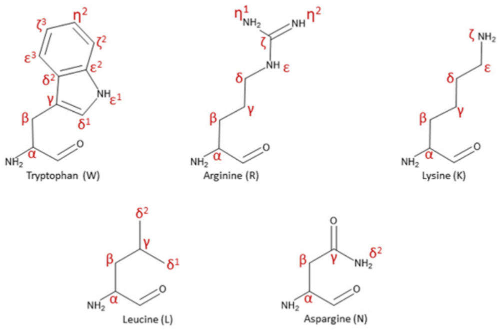

<u>Figure S3</u>: Labelled amino acids.

.

Table S3: Full assignment of c(WWWKKK) according to Figure S2

c(WWWKKK)														
Residue #	Amino acid	Chemical shift (δ)												
1	W - Tryptophan	NHα	CO	α	β	ε1	δ1	γ	δ2	ε3	ζ3	η2	ζ2	ε2
	^{1}H	7.85	-	4.48	2.99/2.86	10.10	7.06	-	-	7.46	7.09	7.17	7.41	-
	$^{13}C/^{15}N$	118.01	173.41	54.70	26.00	129.28	124.45	108.96	127.01	118.48	119.41	122.04	111.98	136.34
2	W - Tryptophan	NHα	CO	α	β	ε1	δ1	γ	δ2	ε3	ζ3	η2	ζ2	ε2
	^{1}H	7.33	-	4.51	3.11/2.87	10.05	6.95	-	-	7.30	7.05	7.15	7.39	-
	$^{13}C/^{15}N$	119.00	172.67	54.55	26.36	130.00	124.76	108.30	127.06	118.30	119.59	122.16	112.04	136.20
3	W - Tryptophan	NHα	CO	α	β	ε1	δ1	γ	δ2	ε3	ζ3	η2	ζ2	ε2
	^{1}H	7.55	-	4.45	3.06/2.76	10.07	6.92	-	-	7.30	7.02	7.11	7.33	-
	$^{13}C/^{15}N$	121.25	172.67	55.19	26.06	129.56	124.19	108.54	126.86	118.30	119.44	122.05	111.94	136.24
4	K -Lysine	NHα	CO	α	β	γ	δ	ε	ζ					
	^{1}H	7.84	-	3.75	1.49/1.67	0.66	1.30/1.58	2.26	7.41	-	-	-	-	-
	$^{13}C/^{15}N$	120.75	173.08	54.90	30.07	21.86	26.19	39.24	96.51	-	-	-	-	-
5	K -Lysine	NHα	CO	α	β	γ	δ	ε	ζ					
	^{1}H	8.03	-	4.10	1.51	1.68/1.50	1.32/1.24	2.88	7.46	-	-	-	-	-
	$^{13}C/^{15}N$	119.47	173.29	53.60	28.83	30.06	22.34	39.43	96.73	-	-	-	-	-
6	K -Lysine	NHα	CO	α	β	γ	δ	ε	ζ					
	^{1}H	7.77	-	3.88	1.22	0.85/0.74	1.33	2.61	7.37	-	-	-	-	-
	$^{13}C/^{15}N$	119.35	173.76	54.55	29.43	21.86	X	39.24	96.52	-	-	-	-	-

Table S4: Full assignment of c(WKWKWK) according to Figure S2.

c(WKWKWK)														
Residue #	Amino acid	Chemical shift (δ)												
1	W - Tryptophan	NHα	CO	α	β	ε1	δ1	γ	δ2	ε3	ζ3	η2	ζ2	ε2
	^{1}H	7.99	-	4.54	3.09	10.17	7.16	-	-	7.52	7.10	7.17	7.37	-
	^{13}C/^{15}N	119.292	173.01	54.66	25.99	129.67	124.35	108.77	127.06	118.37	119.46	122.03	112.04	136.21
2	K -Lysine	NHα	CO	α	β	γ	δ	ε	ζ	-	-	-	-	-
	^{1}H	7.62	-	3.89	1.42	0.67	1.28	2.55	7.35	-	-	-	-	-
	^{13}C/^{15}N	120.24	172.91	54.55	29.59	21.59	26.21	39.25	134.12/95.83	-	-	-	-	-

Table S5: Full assignment of c(WRWRWR) according to Figure S2.

c(WRWRWR)														
Residue #	Amino acid	Chemical shift (δ)												
1	W - Tryptophan	NHα	CO	α	β	ε1	δ1	γ	δ2	ε3	ζ3	η2	ζ2	ε2
	^{1}H	8.07	-	4.54	3.13	10.13	7.16	-	-	7.53	7.10	7.16	7.37	-
	^{13}C/^{15}N	119.43	172.84	54.84	26.04	129.65	124.44	108.78	126.96	118.23	119.39	122.07	111.97	136.23
2	R - Arginine	NHα	CO	α	β	γ	δ	ε	ζ	η1	η2	-	-	-
	^{1}H	7.65	-	3.90	1.41	0.82	2.71	6.77	-	X	X	-	-	-
	^{13}C/^{15}N	119.96	172.88	54.39	27.28	23.71	40.44	84.78	153.71	X	X	-	-	-

Table S6: Full assignment of c(WWWRRR) according to Figure S2.

c(WWWRRR)														
Residue #	Amino acid	Chemical shift (δ)												
	W - Tryptophan	NHα	CO	α	β	ε1	δ1	γ	δ2	ε3	ζ3	η2	ζ2	ε2
1	^{1}H	7.88	-	4.52	2.85/3.04	10.03	7.04	-	-	7.46	7.07	7.15	7.38	-
	$^{13}C/^{15}N$	118.00	173.20	54.68	26.04	129.05	124.44	109.20	126.79	118.31	119.29	122.02	111.89	136.27
	W - Tryptophan	NHα	CO	α	β	ε1	δ1	γ	δ2	ε3	ζ3	η2	ζ2	ε2
2	^{1}H	7.42	-	4.54	2.98/3.03	10.05	7.02	-	-	7.38	7.08	7.16	7.40	-
	$^{13}C/^{15}N$	119.05	172.55	54.36	26.68	130.00	124.76	108.40	127.37	118.30	119.44	122.03	112.02	136.22
	W - Tryptophan	NHα	CO	α	β	ε1	δ1	γ	δ2	ε3	ζ3	η2	ζ2	ε2
3	^{1}H	7.64	-	4.43	2.85/3.05	10.03	6.94	-	-	7.36	7.04	7.12	7.33	-
	$^{13}C/^{15}N$	121.23	173.10	55.49	26.04	129.50	124.27	108.40	126.82	118.17	119.34	122.03	111.87	136.23
	R - Arginine	NHα	CO	α	β	γ	δ	ε	ζ	η1	η2	-	-	-
4	^{1}H	7.94	-	3.72	1.58/1.50	0.74/0.87	2.75/2.73	6.77	-	X	X	-	-	-
	$^{13}C/^{15}N$	120.23	172.89	54.85	26.37	24.11	40.38	85.03	156.63	X	X	-	-	-
	R - Arginine	NHα	CO	α	β	γ	δ	ε	ζ	η1	η2	-	-	-
5	^{1}H	8.15	-	4.18	1.48/1.75	1.49	3.11	7.12	-	X	X	-	-	-
	$^{13}C/^{15}N$	119.02	172.78	53.24	27.98	24.43	40.54	84.97	156.89	X	X	-	-	-
	R - Arginine	NHα	CO	α	β	γ	δ	ε	ζ	η1	η2	-	-	-
6	^{1}H	7.87	-	3.85	1.29/1.24	0.85/0.93	2.78/2.72	6.80	-	X	X	-	-	-
	$^{13}C/^{15}N$	119.50	173.54	54.83	27.01	23.76	40.21	84.97	156.57	X	X	-	-	-

Table S7: Full assignment of c(LWwNKr) according to Figure S2.

c(LWwNKr)														
Residue #	Amino acid	Chemical shift (δ)												
1	L - Leucine	NHα	CO	α	β	γ	δ							
	1H	8.597	-	4.01	1.32	1.43	0.80/0.72	-	-	-	-	-	-	-
	13C/15N	126.00	173.54	53.33	39.17	24.35	22.25/19.98	-	-	-	-	-	-	-
2	W - Tryptophan	NHα	CO	α	β	ε1	δ1	γ	δ2	ε3	ζ3	η2	ζ2	ε2
	1H	7.45	-	4.70	3.09/3.13	10.01	7.10	-	-	7.49	7.07	7.19	7.43	-
	13C/15N	117.50	172.00	53.34	28.21	129.07	124.68	108.88	127.14	118.34	119.17	121.87	113.01	136.17
3	w - (*R*)-Tryptophan	NHα	CO	α	β	ε1	δ1	γ	δ2	ε3	ζ3	η2	ζ2	ε2
	1H	8.35	-	3.90	2.82/2.91	10.00	7.00	-	-	7.36	7.08	7.16	7.40	-
	13C/15N	126.00	174.22	56.72	25.70	129.48	124.58	108.26	126.50	118.15	119.35	122.01	112.05	136.25
4	N - Aspargine	NHα	CO	α	β	γ	δ2							
	1H	7.68	-	4.28	1.70/2.36	-	6.39/7.03	-	-	-	-	-	-	-
	13C/15N	124.00	172.41	49.63	34.71	174.06	110.50	-	-	-	-	-	-	-
5	K - Lysine	NHα	CO	α	β	γ	δ	ε	ζ					
	1H	7.67	-	4.33	1.52/1.72	1.24/1.15	1.52	2.86	X		-	-	-	-
	13C/15N	119.00	172.23	52.56	31.03	21.62	26.24	39.49	X		-	-	-	-
6	r - (*R*)-Arginine	NHα	CO	α	β	γ	δ	ε	ζ	η1	η2			
	1H	8.11	-	4.21	1.60	1.36/1.49	3.08	7.11	-	X	X	-	-	-
	13C/15N	124.00	174.05	53.66	27.34	24.51	40.61	84.49	156.81	X	X	-	-	-
